# Medial frontal cortex: from self-generated action to reflection on one's own performance

**DOI:** 10.1016/j.tics.2009.11.001

**Published:** 2010-01

**Authors:** Richard E. Passingham, Sara L. Bengtsson, Hakwan C. Lau

**Affiliations:** 1 Department of Experimental Psychology, University of Oxford, South Parks Road, Oxford, UK, OX1 3UD; 2 Wellcome Centre for NeuroImaging, University College London, 12 Queen Square, UK, WC1N 3BG; 3 Department of Psychology, Columbia University, 1190 Amsterdam Avenue, New York, NY 10027, USA

## Abstract

It was suggested over 20 years ago that the supplementary motor cortex is involved in self-generated behaviour. Since then, there have been many studies using electrophysiology and brain imaging of the role of the supplementary motor cortex and anterior cingulate cortex. In light of the findings, the proposal that these regions are crucial for self-generated action has recently been challenged. Here, we review the recent literature and argue that the proposal survives the findings. We further argue that it can be generalised to cover reflection on mental states. Finally, we suggest that the pattern of anatomical connections is consistent with the proposal that the medial frontal cortex is crucially involved in self-generated action and self-reflection.

## Self-generated action

The psychologist B.F. Skinner was the first to distinguish between respondent and operant behaviour. In the former, the animal responds to an external stimulus, such as a light. In the latter, the animal operates on the environment and is or is not rewarded. For example, food is presented to a dog and it comes up and eats it (respondent behaviour). Alternately, the dog comes up of its own accord and is given a scratch on the back (operant behaviour). In one case, there is a change in the environment and, in the other, the initial change is in the dog.

Spontaneous actions such as this are ‘self-initiated’ or ‘self-generated’. These terms are used loosely here to include decisions both as to when to act and as to which action to make when there are no external cues to specify the appropriate action [1]. In an early study, Romo and Schultz [2] compared self-initiated and externally triggered movements, and reported that, on average, cells in both the supplementary motor cortex (SMA) and lateral pre-motor cortex fired earlier on self-initiated movements. Okano and Tanji [3] confirmed the fact that such cells could be found in both areas. However, the authors reported that more cells in the SMA (89%) than in the pre-motor cortex (39%) fired mainly or exclusively in the self-initiated task. In the same task, ∼50% of the cells in the SMA fired well in advance of movement compared with only 12% of the cells in the pre-motor cortex. The pattern in the anterior cingulate sulcus is similar to that in the SMA [4].

If this activity is essential for self-initiated movements, interfering with it should reduce or abolish such movements. Thus, monkeys whose pre-SMA and SMA were removed bilaterally, made few self-initiated movements [5]. Notably, the animals were tested in the dark and, hence, there were no visual cues. Lesions to the same regions did not affect externally triggered movement: animals initiated movements relatively normally when they were required to respond to a tone [5]. In the same study, lesions of the cingulate motor areas [6] in the cingulate sulcus also led to a drastic reduction of self-initiated movements [5].

These effects were selective for the medial frontal cortex: lesions of the lateral pre-motor cortex had little effect on the number of self-initiated movements [7], although the movements were less accurate (reflecting perhaps a kinematic impairment) [8]. The same dissociation was shown for sequences of movement when there were no external cues to specify the order of the moves. Halsband and Passingham [9,10] found that monkeys with lesions of the SMA and pre-SMA failed to learn a new sequence of three movements (pull, turn and lift) from memory. By contrast, animals with lateral pre-motor lesions could learn the task without difficulty.

These and other experiments led us to conclude that the SMA and pre-SMA are particularly involved in self-generated movements [10]. This proposal was first made by Eccles [11], and developed by Goldberg [12,13] on the basis of the effects of lesions in patients. However, this proposal has recently been challenged in a commentary by Nachev *et al.* [14]. Several more recent studies using electrophysiology and brain imaging have addressed the role of the SMA and anterior cingulate cortex. Here, we review this literature and argue that the proposal survives the findings and can be generalised to cover reflection on mental states. We also argue that the evidence for anatomical connections is consistent with the proposal.

## Potential challenges

It is first necessary to clarify the proposal. The issue is not whether there are external cues that might trigger a movement, but whether these uniquely specify the response. For example, in one study by Mushiake *et al.* [15] on internally and externally generated sequences of movement, there were keys for the monkeys to respond to. However, in the memory (i.e. internally generated) conditions, the keys did not specify the order in which the monkeys should respond. In the externally specified condition, each key lit up in turn. The finding was that significantly more cells in the SMA were active in the memory than in the visually specified condition, and significantly more cells in the pre-motor cortex in the visually specified than in the memory condition. Similarly in the experiments using sequences by Nakamura *et al.* [16], there were boxes for the monkeys to respond to, but the boxes did not specify the order in which the animal should touch them. There were cells in the pre-SMA that changed their activity during the learning of such sequences.

Given this proviso, the proposal can still be challenged. In the study by Mushiake *et al.* [15], there were cells in both the SMA and pre-motor cortex that fired in both the memory and visually specified conditions. It would be unreasonable to dismiss these cells as being of no functional importance. Indeed, patients with medial lesions that include the SMA show an increase in simple reaction times to a light, perhaps as the result of a lack of preparation for the stimulus [17]. However, monkeys with lesions in the SMA and pre-SMA do not make any more errors than normal on a visual conditional motor task in which colour cues specify which action should be performed [7]. This is in contrast to the severe impairment in monkeys with pre-motor lesions [7].

Furthermore, if the SMA or pre-SMA are inactivated with muscimol, there is no effect on sequences that are specified by visual cues, but a severe effect on sequences that are performed from memory [18]. It is possible to reconcile the electrophysiological and lesion findings if it is supposed that the cells in the pre-motor cortex that fire on memory-guided sequences derive their input from the SMA via interconnections between the two areas [19]. If the pre-SMA and SMA are inactivated, this would abolish such activity in the pre-motor cortex and, thus, this area could not ‘take over’. This hypothesis could be tested by recording in the pre-motor cortex in monkeys with a lesion in the pre-SMA and SMA.

Imaging experiments also show that there is activation in the SMA and pre-SMA, as well as in the cingulate motor areas, during externally triggered movements. In other words, activation of these areas is not confined to self-initiated movements [20,21]. However, it can be shown with time-resolved fMRI that the activation in the pre-SMA and SMA starts earlier when the movements are self-initiated rather than externally triggered [21], and that this is also earlier than in the motor cortex [22]. In other words, activity in the SMA and pre-SMA reflects preparation for the actions.

These experiments made no distinction between the pre-SMA and/or SMA and the cingulate motor areas in the anterior cingulate sulcus. That there is a distinction was shown by Lau *et al.* [23], who used the Libet task [24], and required subjects either to judge the time at which they acted or the time at which they were first aware of their intention to act. There was an enhancement in a cingulate motor area when subjects attended to their movements, and an enhancement in the pre-SMA when they attended to their intention [25]. These results are consistent with the proposal that the pre-SMA and/or SMA and cingulate motor areas are involved together in self-generated action.

In this experiment, the actions were spontaneous in both conditions. However, the usual comparison is between actions that are performed with or without external cues. The problem is that there are four possible experimental confounds in this comparison:

(i) In a self-generated task, human subjects are instructed to initiate or choose between actions in the scanner, and the implication is that they do so randomly. This means that the activations might reflect working memory, as subjects review their last moves. This is indeed a problem in interpreting the results of imaging studies of the role of the prefrontal cortex in generating actions [1]. However, in the experiments by Thaler *et al.* [5] on self-initiated action in monkeys, there was no such constraint on the times at which the animals acted.
(ii) Responding to an external cue can become routine whereas deciding for oneself is not [14]. This is also a problem in interpreting the results of imaging studies on prefrontal cortex. This was shown by Lau *et al.* [26], who found no difference in activation in the prefrontal cortex for self-generated and externally specified actions if the latter were non-routine. However, in the same experiment, there was differential activation in the pre-SMA for the self-generated task, even though the externally specified task was also attentionally demanding.
(iii) It could be argued that the fact that there can be several possible actions in a self-generated task implies that there is a conflict between the actions. Botvinick *et al.* [27] have suggested that, in self-generated tasks, the responses are ‘underdetermined’ and that the activation of medial frontal cortex could be explained as reflecting the monitoring of conflict. Thus, there is activation in the anterior cingulate cortex in conflict trials on the Eriksen flanker task. In these trials, conflict is deliberately introduced by arranging for the peripheral stimuli to specify a response that is different from the one that is specified by the central stimulus.
  Lau *et al.* [28] therefore compared activation on a self-generation task with activation on the Eriksen flanker task. The differential activations lay in the pre-SMA for generation and the anterior cingulate convexity cortex for the flanker task. Nachev *et al.* [29] required subjects to make a saccade to the left or right and introduced conflict by using a cue that specified a change in plan. The peak was in the part of the supplementary eye field as defined by the activation for antisaccades, anterior to the part that is activated for saccades [30]. Although the task involved inhibition, it differed from the Eriksen flanker task in that there were no conflicting visual cues.
(iv) Nachev *et al.* [14] have suggested that the conditions for action are more complex for self-initiated than for externally-specified actions. We agree that talking about actions as being generated by ‘inner cues’ is vague, because the cues are not specified. We also agree with the fact that there are various conditions for action, and that it is necessary to spell them out in detail. We therefore do so in the next section.

## Conditions for action

Some of the conditions for self-generated action include:

(i) Actions can be specified by the time interval that has elapsed, as in the Libet task [24], and there are cells in the pre-SMA and SMA that code for time intervals [31].
(ii) One action can serve as a cue for the next action as in generating a series of actions on the ‘free selection’ task [1], and there are cells in the pre-SMA and SMA that code for specific transitions [32].
(iii) The representation of the goal (or potential outcome) can serve to retrieve the action that is appropriate in achieving that goal, as in the arm raise task [5].
(iv) A change in the goal can serve to retrieve a change in the action. For example, if one type of food is devalued by feeding the animal on it to satiation, the animal shifts to the action that is appropriate for obtaining the other food [33]. In rats, lesions of the medial frontal cortex disrupt this effect [34]. In monkeys, lesions in the anterior cingulate cortex impair the ability of the animals to choose the action that is appropriate for the type of food reward that is presented [35].
(v) Failure to obtain the expected goal or reward can cue a change in the appropriate action, as in the motor reversal task introduced by Chen *et al.* [36]. Monkeys with lesions in the SMA and pre-SMA were slow to reverse [36], as were monkeys with lesions in the anterior cingulate sulcus and convexity cortex [37], but not monkeys with pre-motor lesions [7].

## Evaluation of outcomes

If there are no external cues to specify the appropriate action, the animal must base its decision on an evaluation of the potential outcomes. It is a crucial finding that there are many cells in the anterior cingulate sulcus that encode the value of goals in terms of their probability, payoff and cost [38]. Indeed, there are more such cells in that area than in the orbital frontal cortex. Both the anterior cingulate cortex and orbital frontal cortex are involved in associations with outcomes, but both lesion [39,40] and imaging evidence [41] implicate the orbital frontal cortex in learned associations between stimuli and outcomes and the anterior cingulate cortex in learned associations between actions and outcomes.

The anterior cingulate cortex is involved in switching between actions only where there is no external cue to specify a switch. Thus, there are cells in the anterior cingulate sulcus in monkeys [42] and humans [43] that fire when an action is not followed by the expected reward, so that an alternative action must be performed on the next trial. But these cells do not fire or fire less if there is an external cue to specify the shift. After a surgical excision in the anterior cingulate cortex, human subjects were slow to switch after a reduction in the expected reward.

Given these findings, it is not surprising that there is activity in the anterior cingulate cortex when human subjects do not obtain the expected outcome, as when they make errors [44]. Rushworth *et al.* [45] interpret these results in terms of the evaluation of outcomes, whether positive or negative [46]. An alternative proposal is that the anterior cingulate cortex is involved in the monitoring of conflict, but Botvinick *et al.* [47] accept that this might be subsumed under the more general description of the evaluation of outcomes.

Rushworth *et al.* [48] go on to relate the functions of the anterior cingulate cortex and pre-SMA and/or SMA by suggesting that the pre-SMA and SMA are involved in ‘voluntary’ or self-generated action, and the anterior cingulate cortex in the evaluation of outcomes. If so, it is clear how a change in that evaluation, as in the devaluation paradigm, could lead to a change in action.

## Reflecting on one's own performance

The activations that relate to value when human subjects learn motor rather than stimulus reversals lie in the mid-cingulate cortex (area 24) and the pre-SMA [41]. In the human brain, cingulate area 24 lies posterior and inferior to the paracingulate cortex area 32. Although a ventral area 32 can be identified in the monkey brain, there does not seem to be any homologue of the human dorsal paracingulate cortex [49].

There is evidence that, in the human brain, this area is also involved in the monitoring of outcomes. Bengtsson *et al.* [50] gave subjects the n-back memory task, having told one group that this was a test of intelligence and the other group that the task was being piloted so as to find the optimal parameters. In fact, the task parameters were designed so as to ensure that performance was equated between the two groups. In both groups, as expected, there was activation in the mid-cingulate cortex when they made errors. However, in the group that took the task to be a challenge to their intelligence, there was also activation in the dorsal paracingulate cortex on error trials.

The paracingulate activation was interpreted as relating to reflection on one's own performance. It was notable that the peak of the enhanced activation lay in the same area as the peak when these subjects were specifically required to rate their own performance [50]. The comparison group rated the task parameters. It was argued that errors were of more significance for the group who took the memory test to be a test of intelligence. Whereas monkeys can monitor outcomes, it is unlikely that they can relate them in this way to a self-image.

There is, of course, no way of asking monkeys to describe themselves, but humans can be asked. It is known that, when subjects rate whether particular trait words do or do not apply to themselves, the peak of activation lies in the dorsal paracingulate cortex in a similar location [51]. The suggestion is that in reflecting on their general characteristics, human subjects evaluate their own actions. For example, ‘I can decide whether I am ‘kind’ by reflecting on the actions that I have performed in the past and on my future intentions’.

As already mentioned, when subjects attend to their actions [23] or intentions [25], the activations are in the medial frontal cortex. Furthermore, a multivariate analysis has been used to show that one can read the content of intentions from preparatory activity in the paracingulate and neighbouring medial polar cortex [52]. As suggested by Frith [53], the reason why the paracingulate cortex is also activated when subjects reflect on the mental states of others might be that it is also involved in reflecting on one's own mental states. Two meta-analyses [54,55] suggest that there is a posterior to anterior organization on the medial frontal surface, with the more anterior paracingulate cortex being involved in metacognition, as in reflecting on mental states.

## Extension to other findings

There is a danger that, in propounding an argument, one cherry picks the data that fit, while ignoring other data. It is for this reason that meta-analyses are useful. Paus and Koski [56,57] have carried out two meta-analyses of activations in the medial frontal cortex, and it is clear that regions within the medial frontal cortex can be activated on a variety of tasks. There are two findings in particular that need to be addressed.

The first relates to retrieval from episodic memory. When subjects are required to retrieve memories of actions as opposed to objects, there are activations in the paracingulate as well as the anterior and posterior cingulate cortex. The activations are in the medial frontal cortex when recall of action is contrasted with recall of objects, but on the lateral surface when subjects retrieve memories concerning objects rather than actions [58,59]. In everyday life, these memories might occur spontaneously, although in these experiments they were cued by a brief verbal description. However, the subjects then had to close their eyes for 20 seconds, during which they had recall and re-experience the event [59]. In other words, they generated the details. We suggest that activations on the medial frontal surface reflect memory of action sequences, where one action serves as the cue for the next action.

The second finding is that, when subjects are at rest as opposed to carrying out a cognitive task, there is more activity in the medial frontal cortex, which forms part of the ‘default system’ [60,61]. It has been suggested that activity in the medial frontal cortex reflects ‘self-referential’ processes [62] or ‘mind wandering’ [63]. There are two potential problems with this interpretation. First, the authors simply inferred that the subjects were engaged in spontaneous thoughts. However, in a study by McGuire *et al.* [64], the subjects rated how frequently ‘stimulus-independent thoughts’ occurred, and the activation in the medial frontal cortex was greater the greater the number of such thoughts. The second problem is that, as Gilbert *et al.* [65] point out, spontaneous thoughts could concern the sounds or vibration of the scanner or other aspects of the scanner environment. However, the crucial issue for the present argument is whether these cues uniquely specified the contents of the thoughts. To use an analogy, sentence completion involves cued recall, but in constrained cases, there can be only one word that is suitable whereas in unconstrained cases there might be many; in such cases, the subjects will need to generate possible words themselves [66]. Similarly, an external stimulus, such as a noise, can cue a specific thought (‘there is a loud noise’) as opposed to unconstrained thoughts (such as ‘perhaps the machine is malfunctioning’).

## Anatomical connections

It remains to be shown why it is that self-generated behaviour and monitoring of one's own performance depend on mechanisms on the medial frontal surface. Passingham *et al.* [67] have argued that the key to understanding the function of any particular brain area lies in establishing its ‘connectional fingerprint’; that is, the way in which the pattern of connections differs from that of other areas.

**Figure 1:**
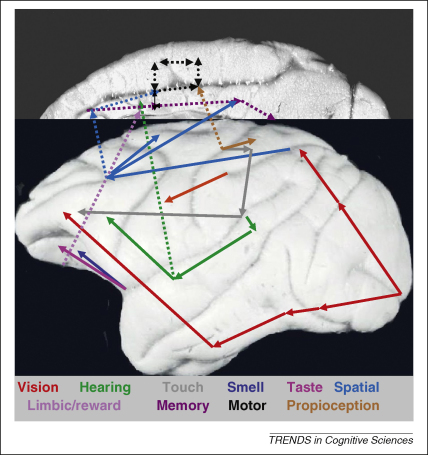
Anatomical connections of the lateral and medial frontal cortex. For references see section on ‘Anatomical Connections’.

Figure 1 compares the connections of the medial and lateral surface of the macaque brain. It is notable that there is only one sensory association area that sends a direct connection to the medial frontal cortex, and this is the superior temporal auditory cortex [68]. It could be that this auditory input accounts for the fact that there is a cingulate motor area in the monkey brain that is involved in spontaneous vocalization [69,70]. Otherwise, the medial frontal cortex receives no direct projections from other sensory association areas [71]. These instead go to the lateral and orbital prefrontal cortex. Whereas the parietal cortex receives information from three senses, the lateral and orbital frontal cortex receives information from all five external senses [72]. The dorsal pre-motor cortex receives visuo-spatial information from the prefrontal cortex [73], whereas the ventral pre-motor cortex receives visual information from the anterior intra-parietal cortex [74].

Whereas the medial frontal cortex receives little direct information about the external world, it has a heavy proprioceptive input from area 3a [75]. At the same time, it is well informed about the internal environment as the result of limbic inputs [76] and, in particular, it is closely interconnected with the amygdala [77,78]. Finally, there are interconnections between the medial motor areas, the cingulate areas processing reward and the cingulate areas that are involved in retrieval from memory [68].

## Concluding remarks

The connections specified above suggest that information about the external world is initially processed on the lateral surface; they are also consistent with the proposal that the integration of information about the actions and internal state of the animal involves structures on the medial frontal surface. However, it is important to be clear about what we are and are not claiming.

First, we are not claiming that information about the self is only processed on the medial surface. We know, for example, that the parietal cortex also receives a proprioceptive input from area 3a [79], and the orbital frontal cortex receives information about the viscera [76]. Our claim only concerns the association of goals with actions and of actions with outcomes.

Second, we are not claiming that, at any one time, one system operates in the brain and that the other does not. We know, for example, that the SMA and the lateral pre-motor cortex are closely interconnected [19], and that cells fire simultaneously in the two areas [2]. This is what would be expected given that the distinction between self-generated and externally guided actions is rarely absolute, but often one of degree. Consider, for example, the verb generation task on which subjects generate verbs from nouns. Here, the noun acts as a prompt but it does not completely specify a particular verb. Instead, the subject has a set of verbs to choose from, and the noun acts to restrict the range of verbs that would be appropriate. When subjects perform this task there is activation both in the lateral prefrontal cortex and the anterior cingulate cortex [80]. Although lesions can dissociate systems artificially, in real life the brain works as a whole. Our proposals are only of any value if they suggest further questions for research. The most crucial ones are outlined in Box 1.

## Box 1 Questions for future research

• Does the degree to which external cues do or do not fully specify the action relate to the degree of activation in the medial frontal cortex?
• Is the activation of medial frontal cortex during episodic recall restricted to the recall of sequences of action and, if so, does it matter whether recall is prompted by external cues?
• Does activation in the ‘default system’ relate to the spontaneous generation of thoughts, irrespective of whether they are about the external environment or about the self?

## References

[1] Passingham R.E., Lau H.C., Pockett S. (2006). Free choice and the human brain. Does Consciousness Cause Behavior?.

[2] Romo R., Schultz W. (1987). Neuronal activity preceding self-initiated or externally timed arm movements in area 6 of monkey cortex. Exp. Brain Res..

[3] Okano K., Tanji J. (1987). Neuronal activity in the primate motor fields of the agranular frontal cortex preceding visually triggered and self-paced movements. Exp. Brain Res..

[4] Shima K. (1991). Two movement-related foci in the primate cingulate cortex observed in signal-triggered and self-paced forelimb movements. J. Neurophysiol..

[5] Thaler D. (1995). The functions of the medial premotor cortex (SMA). I. Simple learned movements. Exp. Brain Res..

[6] Dum R.P., Strick P.L., Vogt B.A., Gabriel M. (1993). Cingulate motor areas. Neurobiology of Cingulate Cortex and Limbic Thalamus.

[7] Passingham R.E. (1993). The Frontal Lobes and Voluntary Action.

[8] Kurata K., Hoffman D.S. (1994). Differential effects of muscimol microinjection into dorsal and ventral aspects of the premotor cortex of monkeys. J. Neurophysiol..

[9] Halsband U., Gantchev G.N. (1987). Higher disturbances of movement in monkeys (*Macaca mulatta*). Motor Control.

[10] Passingham R.E., Porter R. (1987). Two cortical systems for directing movements. Motor Areas of the Cerebral Cortex.

[11] Eccles J.C. (1982). The initiation of voluntary movements by the supplementary motor cortex. Arch. Psychiatry.

[12] Goldberg G. (1985). Supplementary motor area structure and function: review and hypotheses. Behav. Brain Res..

[13] Goldberg G., Perecman E. (1987). From intent to action: evolution and function of the premotor systems of the frontal lobe. The Frontal Lobes Revisited.

[14] Nachev P. (2008). Functional role of the supplementary and pre-supplementary motor areas. Nat. Rev. Neurosci..

[15] Mushiake H. (1991). Neuronal activity in the primate premotor, supplementary, and precentral motor cortex during visually guided and internally determined sequential movements. J. Neurophysiol..

[16] Nakamura K. (1998). Neuronal activity in medial frontal cortex during learning of sequential procedures. J. Neurophysiol..

[17] Viallet F. (1995). Bilateral and side-related reaction time impairments in patients with unilateral cerebral lesions of a medial frontal region involving the supplementary motor area. Neuropsychologia.

[18] Shima K., Tanji J. (1998). Both supplementary and presupplementary motor areas are crucial for the temporal organization of multiple movements. J. Neurophysiol..

[19] Luppino G. (1990). Cortico-cortical connections of two electrophysiologically identified arm representations in the mesial agranular frontal cortex. Exp. Brain Res..

[20] Deiber M.-P. (1999). Mesial motor areas in self-initiated versus externally triggered movements examined with fMRI: effect of movement type and rate. J. Neurophysiol..

[21] Cunnington R. (2002). The preparation and execution of self-initiated and externally-triggered movement: a study of event-related fMRI. Neuroimage.

[22] Cunnington R. (2003). The preparation and readiness for voluntary movement: a high-field event-related fMRI study of the Bereitschafts-BOLD response. Neuroimage.

[23] Lau H.C. (2006). On measuring the perceived onsets of spontaneous actions. J. Neurosci..

[24] Libet B. (1993). Time of conscious intention to act in relation to onset of cerebral activity (readiness-potential). The unconscious initiation of a freely voluntary act. Brain.

[25] Lau H.C. (2004). Attention to intention. Science.

[26] Lau H.C. (2004). Willed action and attention to the selection of action. Neuroimage.

[27] Botvinick M.M. (2004). Conflict monitoring and anterior cingulate cortex: an update. Trends Cogn. Sci..

[28] Lau H. (2006). Dissociating response selection and conflict in the medial frontal surface. Neuroimage.

[29] Nachev P. (2005). Volition and conflict in human medial frontal cortex. Curr. Biol..

[30] Grosbras M.-H. (1999). Anatomical landmark for the supplementary eye fields in human revealed with functional magnetic resonance imaging. Cereb. Cortex.

[31] Mita A. (2009). Interval time coding by neurons in the presupplementary and supplementary motor areas. Nat. Neurosci..

[32] Shima K., Tanji J. (2000). Neuronal activity in the supplementary and presupplementary motor areas for temporal organization of multiple movements. J. Neurophysiol..

[33] Dickinson A. (1996). Bidirectional instrumental conditioning. Q. J. Exp. Psychol. B.

[34] Ostlund S.B., Balleine B.W. (2005). Lesions of medial prefrontal cortex disrupt the acquisition but not the expression of goal-directed learning. J. Neurosci..

[35] Hadland K.A. (2003). The anterior cingulate and reward-guided selection of actions. J. Neurophysiol..

[36] Chen Y. (1995). The functions of the medial premotor cortex (SMA). II. The timing and selection of learned movements. Exp. Brain Res.

[37] Kennerley S.W. (2006). Optimal decision making and the anterior cingulate cortex. Nat. Neurosci..

[38] Kennerley S.W. (2009). Neurons in the frontal lobe encode the value of multiple decision variables. J. Cogn. Neurosci..

[39] Izquierdo A. (2004). Bilateral orbital prefrontal cortex lesions in rhesus monkeys disrupt choices guided by both reward value and reward contingency. J. Neurosci..

[40] Rudebeck P.H. (2008). Frontal cortex subregions play distinct roles in choices between actions and stimuli. J. Neurosci..

[41] Glascher J. (2009). Determining a role for ventromedial prefrontal cortex in encoding action-based value signals during reward-related decision making. Cereb. Cortex.

[42] Shima K., Tanji J. (1998). Role for cingulate motor area cells in voluntary movement selection based on reward. Science.

[43] Williams Z.M. (2004). Human anterior cingulate neurons and the integration of monetary reward with motor responses. Nat. Neurosci..

[44] Yeung N. (2004). The neural basis of error detection: conflict monitoring and the error-related negativity. Psychol. Rev..

[45] Rushworth M.F. (2005). Cognitive neuroscience: resolving conflict in and over the medial frontal cortex. Curr. Biol..

[46] Walton M.E. (2004). Interactions between decision making and performance monitoring within prefrontal cortex. Nat. Neurosci..

[47] Botvinick M.M. (2007). Conflict monitoring and decision making: reconciling two perspectives on anterior cingulate function. Cogn. Affect. Behav. Neurosci..

[48] Rushworth M.F. (2004). Action sets and decisions in the medial frontal cortex. Trends Cogn. Sci..

[49] Ongur D. (2003). Architectonic subdivision of the human orbital and medial prefrontal cortex. J. Comp. Neurol..

[50] Bengtsson S.L. (2009). Motivation to do well enhances responses to errors and self-monitoring. Cereb. Cortex.

[51] Ochsner K.N. (2005). The neural correlates of direct and reflected self-knowledge. Neuroimage.

[52] Haynes J.D. (2007). Reading hidden intentions in the human brain. Curr. Biol..

[53] Frith C. (2002). Attention to action and awareness of other minds. Conscious. Cognition.

[54] Amodio D.M., Frith C.D. (2006). Meeting of minds: the medial frontal cortex and social cognition. Nat. Rev. Neurosci..

[55] Gilbert S.J. (2006). Functional specialization within rostral prefrontal cortex (area 10): a meta-analysis. J. Cogn. Neurosci..

[56] Paus T. (1998). Regional differences in the effects of task difficulty and motor output on blood flow response in the human anterior cingulate cortex: a review of 107 PET activation studies. NeuroReport.

[57] Koski L., Paus T. (2000). Functional connectivity of the anterior cingulate cortex within the human frontal lobe: a brain-mapping meta-analysis. Exp. Brain Res..

[58] Hassabis D. (2007). Using imagination to understand the neural basis of episodic memory. J. Neurosci..

[59] Summerfield J.J. (2009). Cortical midline involvement in autobiographical memory. Neuroimage.

[60] Raichle M.E., Snyder A.Z. (2007). A default mode of brain function: a brief history of an evolving idea. Neuroimage.

[61] Gusnard D.A. (2001). Medial prefrontal cortex and self-referential mental activity: relation to a default mode of brain function. Proc. Natl. Acad. Sci. U. S. A..

[62] Sheline Y.I. (2009). The default mode network and self-referential processes in depression. Proc. Natl. Acad. Sci. U. S. A..

[63] Mason M.F. (2007). Wandering minds: the default network and stimulus-independent thought. Science.

[64] McGuire P.K. (1996). Brain activity during stimulus independent thought. NeuroReport.

[65] Gilbert S.J. (2007). Comment on ‘Wandering minds: the default network and stimulus-independent thought’. Science.

[66] Nathaniel-James D.A., Frith C.D. (2002). The role of the dorsolateral prefrontal cortex: evidence from the effects of contextual constraint in a sentence completion task. Neuroimage.

[67] Passingham R.E. (2002). The anatomical basis of functional localization in the cortex. Nat. Rev. Neurosci..

[68] Barbas H. (1994). Medial prefrontal cortices are unified by common connections with superior temporal cortices and distinguished by input from memory-related areas in the rhesus monkey. J. Comp. Neurol..

[69] Jurgens U. (1970). Cerebral representation of vocalization in the squirrel monkey. Exp. Brain Res..

[70] Jurgens U. (2002). 2-Deoxyglucose uptake during vocalization in the squirrel monkey brain. Behav. Brain Res..

[71] Averbeck B.B., Seo M. (2008). The statistical neuroanatomy of frontal networks in the macaque. PLoS Comput. Biol..

[72] Passingham R.E., Humphreys G.W., Riddoch M.J. (2005). Prefrontal cortex and attention to action. Attention in Action.

[73] Wang Y. (2002). Spatial distribution and density of prefrontal cortical cells projecting to three sectors of the premotor cortex. NeuroReport.

[74] Borra E. (2008). Cortical connections of the macaque anterior intraparietal (AIP) area. Cereb. Cortex.

[75] Jones E.G. (1978). Intracortical connectivity of architectonic fields in the somatic sensory, motor and parietal cortex of monkeys. J. Comp. Neurol..

[76] Carmichael S.T., Price J.L. (1995). Limbic connections of the orbital and medial prefrontal cortex in macaque monkeys. J. Comp. Neurol..

[77] Amaral D.G., Price J.L. (1984). Amygdalo-cortical projections in the monkey (*Macaca fascicularis*). J. Comp. Neurol..

[78] Ghashghaei H.T. (2007). Sequence of information processing for emotions based on the anatomic dialogue between prefrontal cortex and amygdala. NeuroImage.

[79] Huffman K.J., Krubitzer L. (2001). Area 3a: topographic organization and cortical connections in marmoset monkeys. Cereb. Cortex.

[80] Raichle M.E. (1994). Practice-related changes in human brain functional anatomy during non-motor learning. Cereb. Cortex.

